# Global Signal Topography of the Human Brain: A Novel Framework of Functional Connectivity for Psychological and Pathological Investigations

**DOI:** 10.3389/fnhum.2021.644892

**Published:** 2021-03-25

**Authors:** Yujia Ao, Yujie Ouyang, Chengxiao Yang, Yifeng Wang

**Affiliations:** Institute of Brain and Psychological Sciences, Sichuan Normal University, Chengdu, China

**Keywords:** fMRI, global signal topography, functional connectivity, psychopathology, local-global confusion, spatiotemporal integration

## Abstract

The global signal (GS), which was once regarded as a nuisance of functional magnetic resonance imaging, has been proven to convey valuable neural information. This raised the following question: what is a GS represented in local brain regions? In order to answer this question, the GS topography was developed to measure the correlation between global and local signals. It was observed that the GS topography has an intrinsic structure characterized by higher GS correlation in sensory cortices and lower GS correlation in higher-order cortices. The GS topography could be modulated by individual factors, attention-demanding tasks, and conscious states. Furthermore, abnormal GS topography has been uncovered in patients with schizophrenia, major depressive disorder, bipolar disorder, and epilepsy. These findings provide a novel insight into understanding how the GS and local brain signals coactivate to organize information in the human brain under various brain states. Future directions were further discussed, including the local-global confusion embedded in the GS correlation, the integration of spatial information conveyed by the GS, and temporal information recruited by the connection analysis. Overall, a unified psychopathological framework is needed for understanding the GS topography.

## Introduction

In fMRI studies, the global signal (GS), as the grand average of brain signals, is the largest scale of signal integration in the whole brain. It has spurred a widespread debate in the past decade (Fox et al., 2009; Power et al., 2014, 2017). The core issue of the debate is what information the GS preserves (Liu et al., 2017). Early studies considered GS as a major confounding factor in investigating the resting-state network organization (Fox et al., 2009; Murphy et al., 2009), thus widely applying GS linear regression (GSR) to remove the effect of GS from fMRI data prior to network analyses (Macey et al., 2004; Fox et al., 2005; Ciric et al., 2018).

However, significant evidence has uncovered the neurobiological information in the GS, suggesting that GSR may inadvertently discard important neural signals (Fox et al., 2009; Saad et al., 2012; Yeo et al., 2015; Murphy and Fox, 2017; Power et al., 2017). On the one hand, some studies have demonstrated that GS fluctuations do have some influence on and are also influenced by local neural activities in a static or dynamic way, revealing the neural basis of GS (Turchi et al., 2018; Gutierrez-Barragan et al., 2019). On the other hand, the amplitude of GS fluctuations/GS variation (GSV) has been revealed to be associated with mental states, such as vigilance (Wong et al., 2013, 2016), conscious states (Orban et al., 2020; Tanabe et al., 2020), and mental disorder (Zhu et al., 2018). Since once “nuisance” is now a “signal” (Uddin, 2020), the prevailing view is that the GS contains both non-neural and neural information (Murphy and Fox, 2017).

However, the GS is just a single value for illustrating the whole-brain neural activity without considering signals from specific brain regions. Meaningful effects would be inevitably diluted or attenuated by comparing GS fluctuations between groups for the GS intrinsically averaging across correlated and uncorrelated regions (Gotts et al., 2012). Critically, the spatial representation of psychological mechanisms and pathological treatment cannot be provided by GS fluctuations. The relationship between GS and local neural activities may hold the key to unlock the secret of this “catch-all” indicator. In this paper, we reviewed an emerging method called “GS topography,” which reflects the spatial distribution of GS representation, to solve this issue.

## GS Topography, a New Frontier in Neuroimaging

In the human brain, all regions do not work independently but execute psychological functions in a coordinated manner, which has made researchers shift their focus from local neural activities to functional connectivity (FC) to explain the psychological phenomenon in a mutually connected perspective. A seed-based analysis is a classic method of establishing FC by calculating the correlation of time series between selected regions of interest (ROI) and other voxels/regions (Fox and Raichle, 2007). This method relies heavily on the selection of appropriate ROIs, which would be difficult if the underlying psychopathological mechanism is unclear (Nair et al., 2014). This limitation has been partially addressed by some data-driven approaches, such as the principal component analysis and independent component analysis (Biswal et al., 2010).

In face of this limitation, many recent studies have applied global brain connectivity (GBC), a data-driven technique, to illustrate whole-brain connectivity. In the GBC, an *n* × *n r*-value FC map is obtained by calculating correlations between voxels/regions and converted to *z*-value using the Fisher’s *Z* transformation. The weighted GBC (wGBC) for a given voxel/region is defined as the mean *z*-value of correlations between that voxel/region and all other voxels/regions, whereas the unweighted GBC (uGBC) is the count of these correlations over a given threshold (Cole et al., 2010). Therefore, the GBC reflects the overall connection of each voxel/region, providing an unbiased, and non-artificial evaluation of the FC map (Cole et al., 2010).

In a similar vein, the GS topography has been established to measure the correlation between local brain signals and the GS, i.e., GSCORR. After obtaining the GS by averaging signals of all voxels, the GSCORR is measured by calculating temporal correlations between the GS and signal in each voxel. The distribution of GSCORR has been demonstrated to be very similar to the topographies of the uGBC (*r* = 0.96, Zhang et al., 2019) and the wGBC (*r* = 0.88, Scalabrini et al., 2020), indicating that most of the information is consistent among them. Of note, one obvious difference between GSCORR and GBC is that the former contains information of GS. It is suggested that the frequency and phase of GS modulate network states (Scheinost et al., 2016; Gutierrez-Barragan et al., 2019). The amplitude of GS also carries valuable neural information as mentioned above, which influences GS topography directly. Taken together, the GS topography is graced by global information which endows it with unique features. In fact, many recent studies have revealed rich information hidden in the GS topography, making it become a new frontier in psychological and pathological researches.

## Progress of GS Topography

### Intrinsic Architecture of the GS Topography

**FIGURE 1:**
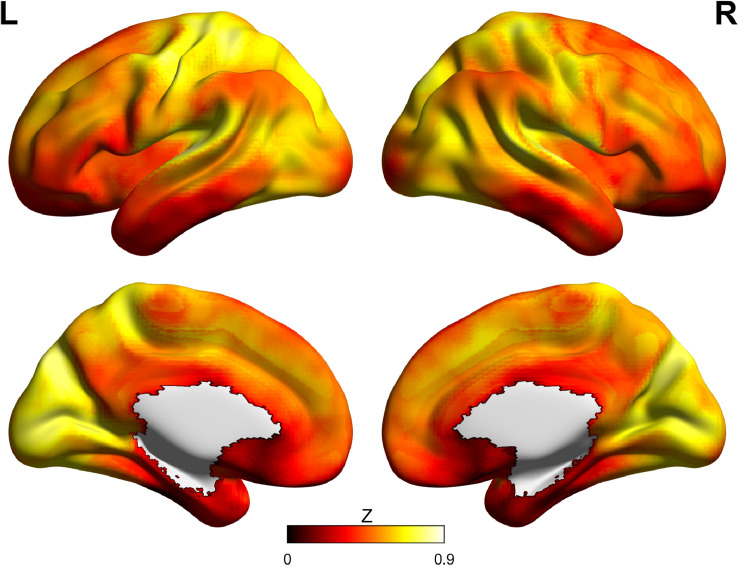
The spatial distribution of Fisher’s *Z* value of GS topography (0.01–0.08 Hz) using a dataset from the Human Connectome Project 100 unrelated subjects (https://db.humanconnectome.org). Higher GSCORR is mainly located in sensory cortices (visual, auditory, and somatosensory regions) and lower GSCORR in higher-order cortices (prefrontal and parietal cortices).

The cortical organization of functional brain networks has been revealed to be largely consistent across resting and various task states, suggesting the existence of an intrinsic architecture of functional networks (Cole et al., 2014, 2016; Gratton et al., 2018). Several studies have discussed the large-scale gradient from sensorimotor to transmodal areas in cortical organization (Mueller et al., 2013; Huntenburg et al., 2017, 2018; Jiang et al., 2020). This gradient cortical organization reflects the sensorimotor-to-transmodal heterogeneities of neurodevelopmental order, FC, and gene expression (Huntenburg et al., 2018). Therefore, it is considered to be the intrinsic anatomical and functional structure of the human brain. As shown in Figure 1, the distribution of GS topography has been revealed to show a similar mode characterized by higher GSCORR in sensory cortices (visual, auditory, and somatosensory regions) and lower GSCORR in higher-order cortices (prefrontal and parietal cortices) in the resting state (Power et al., 2017; Yang et al., 2017; Zhang et al., 2019, 2020; Li et al., 2020). It is suggested that sensory cortices primarily process external stimuli through parallel circuits and networks to ensure cognitive consistency, whereas high-order association cortices integrate sensory inputs into uniform information (Huth et al., 2016; Margulies et al., 2016). Therefore, multisensory inputs activate sensory cortices parallelly, leading to higher levels of correlation across sensory networks, further resulting in a stronger GSCORR. In contrast, fewer shared neural activities would exhibit a relatively weaker GSCORR across association areas (Yang et al., 2017). This hypothesis entails the previous view that the brain’s spatial arrangement follows a global gradient between sensorimotor and transmodal systems. This intrinsic arrangement is considered to be a key feature of the brain to accommodate ever-changing external situations (Huntenburg et al., 2018).

### Psychological Significance of GS Topography

The intrinsic architecture of GS topography raises an important question: Is architecture modulated by a variety of psychopathological states and, if so, how? To answer this question, Li et al. (2019) conducted the canonical correlation analysis (CCA) between principal components derived from the GS topography and those derived from behavioral data. A positive correlation was found between the frontoparietal control network with behavioral outcomes, while a negative correlation was observed between sensorimotor/visual networks and behavioral outcomes. It is worth noting that the positive–negative axis was also found in FC maps (Finn et al., 2015; Smith et al., 2015) wherein the default mode network (DMN) and frontoparietal network contribute most to individual traits but sensory regions contribute few (Smith et al., 2015), indicating that the GS topography and FC map are sensitive to individual factors in different ways.

Another study compared the GS topography between resting-state and seven cognitive tasks (Zhang et al., 2020). Consistent reductions of GSCORR were found in all tasks relative to resting-state, in regions considered to be task-unspecific, including auditory, sensorimotor cortex, and DMN. In contrast, task-specific regions, including the visual cortex and some regions in the frontoparietal network and ventral attention network, exhibited unchanged or a small set of increased GSCORR. Considering all the visual-based and attention-demanding tasks here, the GS topography may be coarsely modulated in sensory and transmodal areas rather than in highly task-specific regions. Alternatively, the sensorimotor and transmodal dichotomous architecture of GS topography may be highly tolerant of cognitive tasks and hard to change. Besides, like the classic functional network, the intrinsic architecture of GS topography is mildly modulated by cognitive tasks, indicating that these two architectures have similar dynamic properties. These hypotheses warrant further investigations. This study also inspires finer investigations on relationships between GSCORR and particular cognitive processes indicated by various methods such as brain activation, brain signal variability, FC, and so on.

Since the GS is closely associated with vigilance, it can be speculated that the GS topography may be modulated by different conscious states. Based on this hypothesis, Tanabe and colleagues tested the GS topography during physiologic, pharmacologic, and pathologic unconscious states in humans and rats. They found that unconsciousness is accompanied by a consistent reduction of GSCORR (Tanabe et al., 2020). Specifically, GSCORR is decreased in the majority of networks in general anesthesia and unresponsive wakefulness syndrome, and in sensory and attention networks in stage 3 of sleep. However, decreased FC within sensory networks with the loss of consciousness has rarely been emphasized in previous FC studies (Larson-Prior et al., 2011; Uehara et al., 2014; Demertzi et al., 2015; Hannawi et al., 2015; Riehl et al., 2017; Golkowski et al., 2019). It seems that altered FC patterns in sensory networks depend more on the GS than specific local connections. This study further suggests that the GS topography is sensitive to general vigilance-based brain states.

Combining these findings, it can be seen that the GS topography is modulated by individual factors, cognitive tasks, and conscious states but that it is not sensitive enough to task details. The lack of task specificity in GS topography may be caused by the dominance of vigilance information from GS, which could be modulated not by overwhelming attention-unrelated information but by attention-demanding tasks (Zhang et al., 2020). Alternatively, the task specificity of GS topography may be determined by the network construction method. Similarly, inconsistent task-specific FC maps were reported with different network construction methods (Cole et al., 2016; Gratton et al., 2018; Di and Biswal, 2019; Sasai et al., 2021). Therefore, more investigations are needed to clarify the cognitive and state characteristics of GS topography.

### Pathological Significance of the GS Topography

As shown in Table 1, resting-state fMRI studies have revealed altered GS topography in several psychiatric and neurological disorders. Similar to abnormal brain regions in classic FC analysis (Kaiser et al., 2015; Li et al., 2018; Syan et al., 2018; Yan et al., 2019; Zovetti et al., 2020), altered GS topography is mainly located in higher-order association networks (such as the DMN, limbic affective network, frontoparietal network, and salience network), with a relatively small part in the sensorimotor network. However, there may be different pathological mechanisms indicated by GS topography and classic FC. As mentioned in section “Psychological significance of GS topography,” the GS topography is sensitive to general arousal-based or attention-demanding brain functions, which may be caused by the neural generator of GS in the basal forebrain (Turchi et al., 2018). Since the basal forebrain is modulated by the locus coeruleus–noradrenergic system (España and Berridge, 2006), we consider the abnormality of GS topography could be traced to the disrupted locus coeruleus–noradrenergic system. Indeed, the locus coeruleus–noradrenergic system has been demonstrated to be associated with various mental disorders, indicating the potential relationship between GS topography, and the locus coeruleus–noradrenergic system (Baumann et al., 1999; Anticevic et al., 2014; Kuffel et al., 2014). If so, the GS topography may provide an appropriate biomarker for medical treatment of attention, arousal, or conscious dysfunction associated with various mental disorders.

**TABLE 1:** Altered GS topography in mental diseases.

References	Type of disease	Sample	Abnormal brain regions
Yang et al., 2017	SCZ	Dataset 1: 90 patients 90 HC Dataset 2: 71 patients 74 HC	Decreased GSCORR in sensory regions Increased GSCORR in association regions
Wang et al., 2019	SCZ	39 early-onset patients 31 HC	Static: Decreased GSCORR in right superior temporal gyrus Dynamic: Decreased GSCORR in right middle temporal gyrus, left middle temporal Gyrus, left precuneus, and left calcarine. Increased GSCORR in left cerebellum crus 1, left middle cingulate gyrus, right putamen, right precuneus, and right supramarginal gyrus
Wang et al., 2020	SCZ	39 early-onset patients 31 HC	GS topography in 0.01–0.027 Hz: sensory network GS topography in 0.027–0.073 Hz: DMN
Han et al., 2019	MDD	63 patients 63 HC	Static: decreased GSCORR in the left middle temporal gyrus, bilateral parahippocampal gyrus, bilateral hippocampus gyrus, and right fusiform gyrus Dynamic: increased standard deviation of the dynamic GSCORR in right parahippocampal gyrus, right hippocampus gyrus, and right ventromedial prefrontal cortex
Scalabrini et al., 2020	MDD	49 patients 50 HCs	Increased GSCORR in default mode network
Zhang et al., 2019	BD	99 patients (30 in the manic phase, 35 in the depressive phase, and 34 in euthymic phase) 64 HC	Depressed phase: increased GSCORR in left hippocampus, parahippocampus, and fusiform area. Manic phase: increased GSCORR in bilateral motor cortex Euthymic phase: decreased GSCORR in pregenual anterior cingulate cortex
Li et al., 2020	Epilepsy	127 patients in IGE-GTCS 114 patients in TLE 161 HC	IGE-GTCS: decreased GSCORR in para/hippocampus, cerebellum, midbrain tegmentum, and calcarine gyrus. Increased GSCORR in orbital frontal cortex and medial frontal cortex. TLE: decreased GSCORR in para/hippocampus, midbrain tegmentum, and middle temporal gyrus. Increased GSCORR in orbital frontal cortex.

*HC, Healthy Controls; IGE-GTCS, Idiopathic Generalized Epilepsy with Generalized Tonic–clonic Seizures; and TLE, Temporal Lobe Epilepsy.*

### GS Topography: The “Spatiotemporal Psychopathology”

Many studies have found specific spatial alternations of GS topography in various psychological and pathological states. A few studies, however, have concerned the temporal aspect of GS topography, such as the temporal dynamics and frequency characteristics (Scheinost et al., 2016; Wong et al., 2016; Gutierrez-Barragan et al., 2019). Each value of the GS topography measures the temporal co-activation of local and global neural activities, supporting the idea that symptoms of psychopathology are not only caused by disrupted function in local brain regions but are also driven by a global spatiotemporal organization (Scalabrini et al., 2020). Based on this idea, the “spatiotemporal psychopathology” was put forward to link the global organization of the human brain to psychopathological symptoms (Northoff, 2016a,b). This concept shifts the focus from internal or external stimuli and specifical brain functions to the spatiotemporal organization, such as whole-brain functional networks, global-to-local neural activities, and the profile of full frequency power spectrum (Northoff et al., 2020). Overall, the spatiotempral organization of GS topography may play an important role to uncover valuable neural information in GS topograhy, and it may provide a solution to some important questions in psychopathological investigations, including consciousness, self-reference processing, and so on.

## Core Issues and Future Directions

### Understanding the Relationship Between “Local Signal” and “Global Signal”

Although the GS topography seems like a promising index and perspective to solve psychological or pathological problems, there is still a lot to discover underground, just like the GS. A basic issue underlying the GS topography is the relationship between local signal and GS, or rather to say, the local-global confusion. Human brain is a widely connected complex system. The signal within one node, not only represents local neural activity but also contains complex interactions with other nodes. As shown in the above sections, local signal and GS contain each other in the GS topography. This local-global confusion is partially distinguished by comparing results with or without GSR. For instance, a recent study examined the effect of GSR on GSCORR in the DMN (Scalabrini et al., 2020). It has been found that after GSR, significant differences of intra-DMN connectivity largely disappeared between patients with major depressive disorder and healthy controls. It suggests that the meaningful pathological information lies in the global activity, and the DMN activity is strongly influenced and shaped by the GS. However, the causal influences of GS on DMN and local neural activities, or vice versa, have not been examined yet. The effective connectivity (e.g., dynamic causal modeling and Granger causality analysis) may describe the bidirectional influences that global exerts over local or vice versa. All in all, further investigations are needed to explain the independence and interaction between the local and GS.

### Integrating Spatial and Temporal Dimensions

Because the GS topography is established by a temporal correlation between the spatial average of whole-brain signals (GS) and signals in each voxel, it inherently integrates spatial and temporal dimensions. Besides the spatially local-global confusion, the GS topography is temporally limited in the low-frequency range (usually < 0.25 Hz) due to the low sampling rate of fMRI. Although a recent study has tested the relationship between the GS of fMRI and that of electroencephalograph (Huang et al., 2019), high-frequency GS topography has not been studied yet based on techniques with high sampling rates. It is a core mission of GS topography to integrate spatial signals from local to global and temporal signals from low frequency to high frequency. Multimodal approaches such as fMRI, EEG, and other techniques, and multi-index approaches combining amplitude, phase, and frequency are essential to integrate spatial and temporal dimensions in future studies.

## Conclusion

The GS topography describes brain networks from a global-local relationship perspective, providing an unbiased evaluation of the cortical functional organization. Valuable information in the GS topography was uncovered in various situations, such as different conscious states, cognitive tasks, and brain disorders, shedding new light on the psychopathological theory. Some essential issues such as the local-global confusion and the integration of spatiotemporal information are to be resolved in order to clarify the psychological, physiological, and pathological significances of GS topography. A spate of recent studies suggests that GS topography is becoming the next frontier of neuroimaging research.

## Author Contributions

YA wrote the first draft and reviewed the manuscript. YO and CY were instrumental in its improvement. YW provided invaluable guidance throughout its preparation and approved the final version of the manuscript. All authors contributed to the article and approved the submitted version.

## Conflict of Interest

The authors declare that the research was conducted in the absence of any commercial or financial relationships that could be construed as a potential conflict of interest.
